# Attention-Driven CNNs as a Strong Default for HER2 Prediction from DCE-MRI: A Comparison with Transformer Architectures

**DOI:** 10.3390/bioengineering13070788

**Published:** 2026-07-08

**Authors:** Naomi Fridman, Anat Goldstein

**Affiliations:** Department of Industrial Engineering, Ariel University, Ariel 40700, Israel; anatgo@ariel.ac.il

**Keywords:** deep learning, HER2, breast cancer, DCE-MRI, attention mechanisms, convolutional neural networks, medical image analysis

## Abstract

**Background:** HER2 status guides targeted therapy in breast cancer but is currently determined by invasive biopsy. Imaging-based HER2 prediction from dynamic contrast-enhanced MRI (DCE-MRI) could provide a non-invasive adjunct decision-support signal, but published models are typically single-center with heterogeneous preprocessing that limits reproducibility. **Methods:** We trained a Triple-Head Dual-Attention ResNet (THDA-ResNet) that processes three DCE phases (pre-contrast, early post-contrast, and late post-contrast) on the multicenter BreastDCEDL dataset (*n* = 1149, I-SPY trials), and we compared it with Vision Transformer (ViT) and Convolutional Vision Transformer (CvT) baselines, all ImageNet-pretrained. We benchmarked 14 preprocessing strategies, with and without N4 bias-field correction. External validation used the independent BreastDCEDL_AMBL cohort (43 lesions). AUC confidence intervals used stratified bootstrap; model comparisons used DeLong’s test. **Results:** THDA-ResNet achieved the highest AUC, 0.74 (95% CI 0.65–0.83), versus 0.66 for ViT and 0.63 for CvT, with the advantage reaching borderline significance over CvT (p=0.054) and not significant over ViT (p=0.14). At a threshold of 0.7, it retained discrimination (sensitivity 0.41, specificity 0.86), while transformers collapsed to near-trivial classifiers. External AUC was 0.66 (0.49–0.81). N4 correction did not improve performance. **Conclusions:** Attention-driven CNNs are a strong default for HER2 prediction from DCE-MRI on medium-sized cohorts, and N4 correction can be omitted, simplifying the pipeline.

## 1. Introduction

Breast cancer is the most frequently diagnosed cancer in women and a major cause of cancer mortality worldwide [1]. Dynamic contrast-enhanced MRI (DCE-MRI) is central to diagnosis and management, providing high soft-tissue contrast and functional information on contrast kinetics that reflect underlying tumor biology [2]. Molecular subtyping, particularly the assessment of human epidermal growth factor receptor 2 (HER2) status, is critical for guiding targeted therapies [3,4]. However, standard HER2 assessment via biopsy is invasive and subject to sampling bias. A reliable imaging-based method for HER2 prediction from DCE-MRI could improve diagnostic workflow, reduce patient burden, and enable earlier treatment planning.

While some radiomics and deep learning studies have shown promise, most HER2 prediction models are trained and evaluated on small, single-center MRI datasets, frequently using handcrafted features and lacking demonstration of generalizability [5,6]. Models such as CNNs [7,8] and, more recently, Vision Transformers (ViTs) and Convolutional Vision Transformers (CvTs) [9,10,11,12,13] have achieved strong results in medical image classification. Nonetheless, a comparative evaluation of these modern architectures for HER2 prediction from DCE-MRI in a multicenter setting has not been systematically conducted.

Dual-attention ResNet architectures, which incorporate both channel and spatial attention mechanisms, have demonstrated performance gains for diverse clinical imaging tasks including histopathology classification, denoising, and image quality assessment [14,15,16,17,18]. Attention modules allow models to adaptively focus on the most informative features and spatial regions, which is especially valuable in heterogeneous medical images.

An important yet often overlooked challenge in applying deep learning to DCE-MRI is preprocessing of high-dynamic-range MR signal (typically 12–16 bit, with voxel intensities often exceeding 3000–12,000) into a numerically stable range for modern backbones [19]. MR intensity distributions are highly skewed and long-tailed, so mapping 16-bit DICOM intensities either to floating-point [0,1] or to 8-bit integer [0,255] can compress dynamic range and distort contrast relationships between voxels, slices, and temporal phases [19,20]. We systematically evaluate alternative clipping and normalization strategies for DCE-MRI, both with and without N4 bias-field correction, to assess how these choices influence the predictive performance of deep learning models for HER2 status.

To enable robust benchmarking, we trained and evaluated models on the BreastDCEDL dataset [11], comprising 1149 pre-treatment 3D DCE-MRI scans from the I-SPY trials [21,22,23], harmonized and converted to standardized NIfTI volumes with unified annotations. This multicenter dataset enables systematic comparison of state-of-the-art architectures (ViT, CvT, Triple-Head Dual-Attention ResNet) for HER2 prediction using consistent protocols. For external validation, we evaluated the trained models on the independent single-center BreastDCEDL_AMBL dataset [24], which differs from I-SPY in scanner vendor, imaging protocol, and patient population.

Overall, this work leverages a multicenter, standardized dataset to systematically compare preprocessing strategies and leading deep learning architectures for DCE-MRI-based HER2 prediction, providing evidence for cross-institutional generalizability and practical insights for clinical implementation.

## 2. Related Work

### 2.1. Radiomics Approaches

Early MRI-based efforts to predict HER2 status relied on handcrafted radiomic features combined with classical machine-learning models. Zhou et al. [25] performed radiomics-based HER2 classification in a single-center cohort of 306 patients using multiparametric MRI, achieving a validation AUC of 0.81. Xu et al. [26] analyzed 214 invasive ductal carcinoma patients with semi-automatically segmented multiparametric MRI; their radiomics-only model achieved AUC 0.84, improving to 0.87 when combined with Ki-67 and histologic grade. Li et al. [27] reported substantially lower performance (AUC 0.65) on the multicenter TCGA breast MRI dataset (84 cases), underscoring the difficulty of maintaining discrimination across heterogeneous scanners and protocols. These radiomics studies demonstrate that MRI encodes information relevant to HER2 status, but they are constrained by small, often single-center cohorts and limited generalizability.

### 2.2. Deep Learning Approaches

Antropova et al. [28] used pretrained CNNs as feature extractors with an SVM classifier for benign–malignant classification. Chen et al. [29] developed a 3D pipeline fusing features from a video-pretrained ConvNet with handcrafted radiomics, achieving AUC 0.63 on I-SPY2 and 0.56 on I-SPY1, highlighting the challenge of cross-cohort generalization. Ha et al. [30] trained ResNet on first post-contrast images (*n* = 216, single institution), achieving macro-average AUC 0.85 for molecular subtype classification. Shiri et al. [31] evaluated 3D ResNet-like architectures on the public Duke dataset (*n* = 922) using four DCE-MRI time points as input channels; their best model (SEResNext101) achieved AUC 0.70 for HER2 prediction.

### 2.3. Transformer-Based and Attention-Driven Architectures

Zhang et al. [32] proposed a Vision Transformer for distinguishing HER2-zero from HER2-low and HER2-positive cases (*n* = 879, single-institution training cohort with two external validation cohorts), achieving AUC 0.73 and 0.71 on the two external test sets. Wong et al. [33] employed a large-scale self-supervised foundation model trained on 3564 patients across five centers, the largest deep learning study for HER2 prediction to date; the model was used as a feature extractor with XGBoost for three-class HER2 classification and achieved macro-average AUCs of 0.83 and 0.86 in two external validation cohorts. Recent systematic reviews have compared these architectures directly: Takahashi et al. [34] reviewed CNNs and ViTs across medical imaging tasks and reported that ViTs generally require larger training datasets and pretraining to match or exceed CNNs, while Kim et al. [35] reviewed hybrid CNN–ViT architectures that combine the local feature extraction of CNNs with the global context modeling of transformers.

### 2.4. Dual-Attention Mechanisms for Medical Imaging

Dual-attention ResNet architectures, which integrate both channel and spatial attention mechanisms, have demonstrated notable performance gains across a wide range of clinical imaging tasks, including histopathology classification, denoising, and image quality assessment [14,15,16,17,18]. Attention modules enable networks to adaptively emphasize the most informative feature maps and spatial regions, an essential property for modeling tumor heterogeneity in breast DCE-MRI. Despite these demonstrated advantages, dual-attention architectures have not been systematically investigated in the context of HER2 status prediction from DCE-MRI.

### 2.5. Preprocessing Strategies and Normalization Methods

Preprocessing is critical for MRI radiomics and deep learning. Typical steps include N4 bias-field correction [20,36], resampling to a common voxel spacing, and intensity normalization. Recent HER2 prediction studies illustrate pipeline variability: Wong et al. [33] applied N4 correction (50 iterations, convergence threshold 0.001), resampling to $1\times 1\times 1\;{\mathrm{mm}}^{3}$, and z-score normalization, while Shiri et al. [31] used default N4 parameters and min–max normalization without resampling. To assess the effect of these choices, we systematically benchmark multiple normalization strategies in the present work.

### 2.6. Summary and Research Gaps

Prior work on MRI-based HER2 prediction has evolved from handcrafted radiomics to CNNs, transformers, and attention-based architectures, but reported performance for binary HER2 status typically remains modest and drops further under external validation [25,26,27,29,31,33]. Most studies are limited by relatively small, often single-center cohorts and heterogeneous preprocessing pipelines, making it difficult to compare methods or assess generalizability across scanners and institutions. A unified benchmark that compares modern architectures under harmonized preprocessing, and a controlled analysis of how N4-based versus raw-intensity workflows affect deep learning behavior across sites, are still lacking. These gaps motivate the present multicenter evaluation of CNN, transformer, and dual-attention architectures with rigorous external validation.

## 3. Materials and Methods

### 3.1. Datasets

#### 3.1.1. Internal Cohort: BreastDCEDL (I-SPY Trials)

The internal cohort included 1149 breast cancer patients from the multicenter BreastDCEDL dataset [11], derived from the I-SPY 1 and I-SPY 2 clinical trials [21,22,23]. BreastDCEDL is a deep-learning-ready resource that aggregates pre-treatment 3D DCE-MRI scans across the I-SPY participating sites, harmonized into standardized NIfTI volumes with unified annotations and a predefined train/validation/test benchmark split. We used this benchmark split throughout: training (*n* = 885, 77.0%), validation (*n* = 132, 11.5%), and testing (*n* = 132, 11.5%). Distributions of age, hormone receptor (HR) and HER2 status, race, and pathologic complete response (pCR) were well balanced across splits (Table 1).

#### 3.1.2. External Cohort: BreastDCEDL_AMBL

The external cohort was the public BreastDCEDL_AMBL dataset [24], derived from the Advanced-MRI-Breast-Lesions (AMBL) collection on The Cancer Imaging Archive [22]. The AMBL collection is a single-institutional, retrospective dataset of 632 breast MRI imaging sessions acquired at Sheba Medical Center between 2018 and 2021 on a 1.5 T MR system. We used the T1-weighted DCE sequence, which contains five temporal acquisitions (one pre-contrast and four post-contrast) [22]. Lesion segmentations in the source collection were generated by a certified radiologist with 23 years of experience, who manually delineated all enhancing or suspicious regions reported in the radiological reports; the delineations were then refined by automatic thresholding, retaining pixels whose intensity in the first or second subtraction image (post-contrast minus pre-contrast) exceeded a threshold of 75 [22].

The BreastDCEDL_AMBL release [24] curates this collection into a deep-learning-ready format and contains 88 patients with 132 annotated lesions. HER2 status was available for 42 of those patients (43 lesions), which constituted our external HER2 test cohort. We used the segmentation masks provided in the source collection without any additional manual delineation. AMBL differs from I-SPY in scanner vendor, field strength, imaging protocol, and patient population, and was used strictly as a held-out external test set: no training, fine-tuning, or hyperparameter adjustment was performed on AMBL data.

#### 3.1.3. Input Construction

For each patient, we used three temporal phases as input to the network: pre-contrast, early post-contrast, and late post-contrast. These phases reflect the standard kinetic interpretation of DCE-MRI, in which the initial wash-in slope reflects tumor vascular perfusion and permeability, while the late-phase plateau or washout pattern is associated with tumor angiogenesis and microvascular heterogeneity [2]. The specific acquisitions corresponding to these phases depend on the imaging protocol of each site, particularly the temporal resolution between consecutive post-contrast acquisitions, and were therefore selected differently for the two cohorts.

For the I-SPY cohort, the three phases were selected by the expert radiologists who analyzed the original I-SPY studies and generated the tumor segmentation masks; the corresponding DICOM acquisition indices are encoded in the I-SPY trial metadata and released as part of the curated BreastDCEDL dataset [11]. Our pipeline relies only on these publicly released indices and does not require any additional manual selection.

For the AMBL cohort, no radiologist-curated phase indices are provided in the source dataset. Each AMBL examination consists of five temporal acquisitions (one pre-contrast and four post-contrast phases) acquired within a span of approximately ten minutes, which is a denser temporal sampling than I-SPY. We selected acquisition 0 as pre-contrast, acquisition 1 as early post-contrast (whose time interval from pre-contrast is comparable to that of the I-SPY early post-contrast phase), and the last acquisition (acquisition 4) as late post-contrast (representing the latest available washout-phase image in the protocol). The same selection rule was applied to all AMBL patients.

#### 3.1.4. Slice Selection

For each examination, the model was applied to the subset of axial slices that intersected the tumor. In the present work the tumor-bearing slices were identified using the segmentation masks released with each dataset (BreastDCEDL for I-SPY, BreastDCEDL_AMBL for the external cohort); because these masks were used purely to localize which slices contained tumor and were not consumed as a model input, segmentation accuracy is not a strict requirement for the pipeline. A fully automated pipeline can be obtained by replacing the released masks with an off-the-shelf breast lesion segmenter, such as the U-Net ensemble of Khaled et al. [37]; even a coarse segmentation is sufficient because only the slice index is used.

### 3.2. DCE-MRI Preprocessing and Normalization

DCE-MRI intensities exhibit long-tailed distributions with voxel values often exceeding 3000–12,000 units, whereas pretrained backbones expect inputs in [0,1]. We systematically evaluated multiple normalization strategies to assess their impact on HER2 prediction performance.

Bias-field correction.

**Figure 1:**
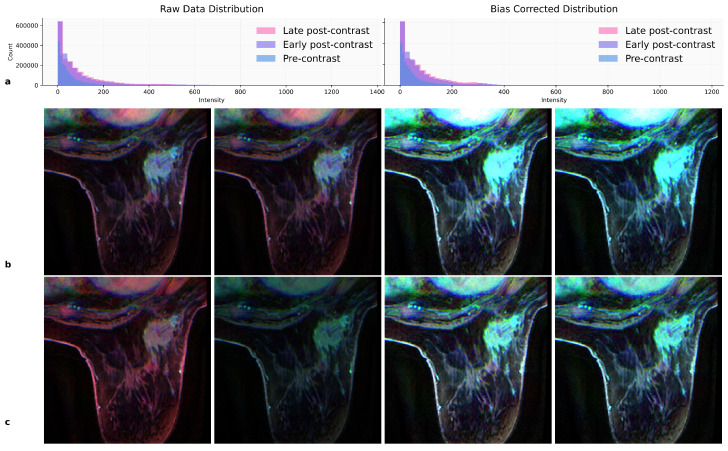
N4 bias-field correction and normalization strategies for DCE-MRI preprocessing. (**a**) Intensity distributions: Raw data (**left**) and N4 bias-corrected data (**right**) across three temporal phases (pre-contrast, early post-contrast, late post-contrast), with 0.99 quantile threshold marked. (**b**) Raw DCE-MRI with four normalization methods: Per-channel min–max, global min–max, per-channel $q_{0.99}$ clipping then min–max, global $q_{0.99}$ clipping then min–max. (**c**) N4 bias-corrected DCE-MRI with identical normalization strategies. RGB channels: Red = pre-contrast, green = early post-contrast, blue = late post-contrast. Slice 10.

We optionally applied N4ITK bias-field correction [20] to reduce low-frequency intensity inhomogeneities (Figure 1a).

Min–max normalization.

We compared per-slice normalization, $x^{\prime }=\left(x-\min\left(x_{\mathrm{slice}}\right)\right)/\left(\max\left(x_{\mathrm{slice}}\right)-\min\left(x_{\mathrm{slice}}\right)\right)$, against global normalization using volume-wide extrema, $x^{\prime }=\left(x-\min\left(x_{\mathrm{vol}}\right)\right)/\left(\max\left(x_{\mathrm{vol}}\right)-\min\left(x_{\mathrm{vol}}\right)\right)$.

Quantile clipping.

To mitigate dynamic-range compression from outliers, we evaluated lower clipping at the 10th percentile, $x^{\prime }=\max\left(x-q_{0.10},0\right)/\left(\max\left(x_{\mathrm{vol}}\right)-q_{0.10}\right)$, and upper clipping at the 95th, 98th, and 99th percentiles, e.g., $x^{\prime }=\min\left(x,q_{0.95}\right)/q_{0.95}$. Both were applied per-slice or globally. Each strategy was tested with and without N4 correction using THDA-ResNet and the transformer baselines (CvT [10], ViT [9]). Figure 1 visualizes preprocessing effects.

### 3.3. Deep Learning Modeling

The three selected temporal phases were stacked into a three-channel (RGB) image following the BreastDCEDL approach [11]: pre-contrast in the red channel, early post-contrast in the green channel, and late post-contrast in the blue channel. Phase selection followed the procedure described above.

#### 3.3.1. Triple-Head Dual-Attention ResNet Architecture

**Figure 2:**
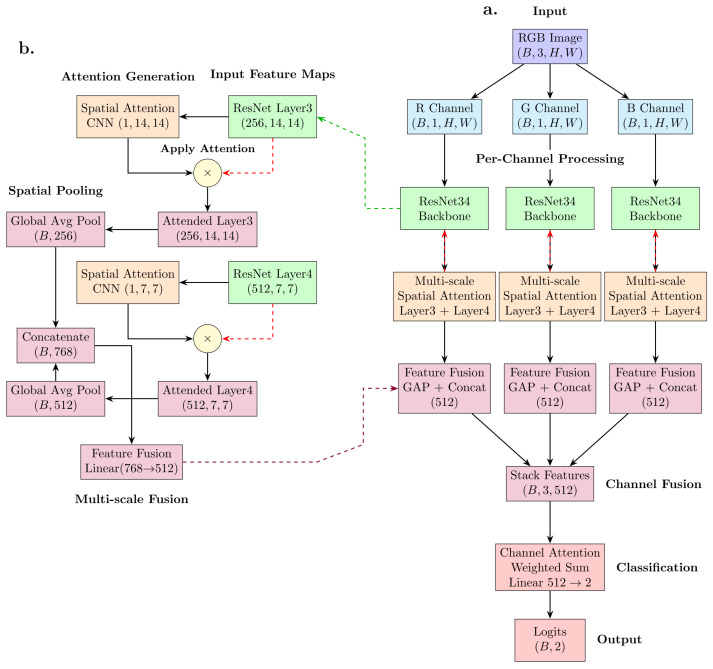
Triple-Head Dual-Attention ResNet architecture for DCE-MRI analysis. (**a**) Overall architecture showing per-channel processing of RGB-fused temporal phases through a single shared-weight ResNet34 backbone with multi-scale spatial attention mechanisms, followed by channel fusion and classification. (**b**) Multi-scale spatial attention module detail demonstrating feature extraction from ResNet Layer3 and Layer4, spatial attention generation, element-wise multiplication for feature refinement, and multi-scale fusion through global average pooling and concatenation.

The proposed model, Triple-Head Dual-Attention ResNet (THDA-ResNet), is a CNN designed for quantitative analysis of breast DCE-MRI. As illustrated in Figure 2, the network processes three temporal phases—pre-contrast, early post-contrast, and late post-contrast—by channel-wise routing each through a single, weight-shared ResNet34 backbone initialized with ImageNet-pretrained weights, so that the same encoder (and its appended attention modules) is applied to all three phases. Multi-scale spatial features are extracted at intermediate layers (Layer 3 and Layer 4), with each branch outputting feature maps of sizes 14×14×256 and 7×7×512, respectively.

Specialized attention modules are appended to both intermediate-layer and Layer4 outputs. These modules consist of stacked convolutional and batch-normalization layers followed by rectified linear unit non-linearity, dropout, and sigmoid gating. The resulting spatial attention maps softly gate their corresponding feature maps through element-wise multiplication, isolating anatomically salient regions. An explicit edge-suppression step down-weights border pixels to 30% of their original value, mitigating border artifacts common in medical imaging.

**Figure 3:**
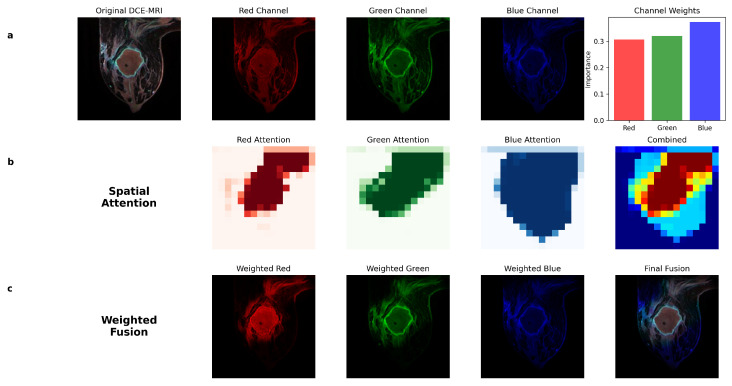
Triple-Head Dual-Attention ResNet mechanism visualization for DCE-MRI temporal fusion. (**a**) Input decomposition showing the original RGB-fused DCE-MRI image and its constituent channels (red: pre-contrast, green: early post-contrast, blue: late post-contrast), with learned channel-attention weights indicating relative importance of each temporal phase. (**b**) Spatial attention maps generated by the multi-scale attention module (Layer3, 14×14 resolution) for each temporal channel, highlighting tumor-relevant regions; combined spatial attention demonstrates consensus across temporal phases. (**c**) Weighted fusion process showing attention-modulated individual channels and their integration into the final fused representation.

Attention-refined multi-scale features are globally pooled and concatenated, capturing both fine and coarse spatial context. The resulting vectors from all three temporal branches are stacked and processed via an adaptive channel-attention mechanism employing a softmax operation, enabling dynamic weighting and aggregation of features from all temporal phases (Figure 3). The fused feature vector undergoes layer normalization and dropout regularization prior to classification via a fully connected output layer. All attention-module weights are initialized using Kaiming normalization [38]. The framework is implemented in PyTorch 2.12.1 and trained end-to-end using AdamW and cross-entropy loss.

#### 3.3.2. Comparative Architectures

For comparative evaluation, we tested two transformer-based architectures: Convolutional Vision Transformer (CvT) [10] and Vision Transformer (ViT) [9]. ViT was selected as the foundational transformer architecture for image classification [9]. CvT was chosen as a representative hybrid architecture that integrates convolutional operations into the transformer framework [10]. All three models were trained under matched conditions, differing only where the architecture required it. They shared the same training/validation/test split, preprocessing pipeline, input size (224×224), optimizer (AdamW), loss function (cross-entropy), batch size (16), ImageNet pretraining, and patient-level median aggregation. THDA-ResNet used a ResNet34 backbone (torchvision ImageNet weights), ViT used ViT-MAE-Base (facebook/vit-mae-base, self-supervised MAE pretraining), and CvT used CvT-13 (microsoft/cvt-13). The learning rate, learning-rate schedule, and dropout were set per architecture, because transformer fine-tuning requires substantially lower learning rates than the CNN; applying the CNN’s learning rate to the transformers destabilized their fine-tuning. The complete set of training and implementation hyperparameters for the three models is given in Table 2.

**Figure 4:**
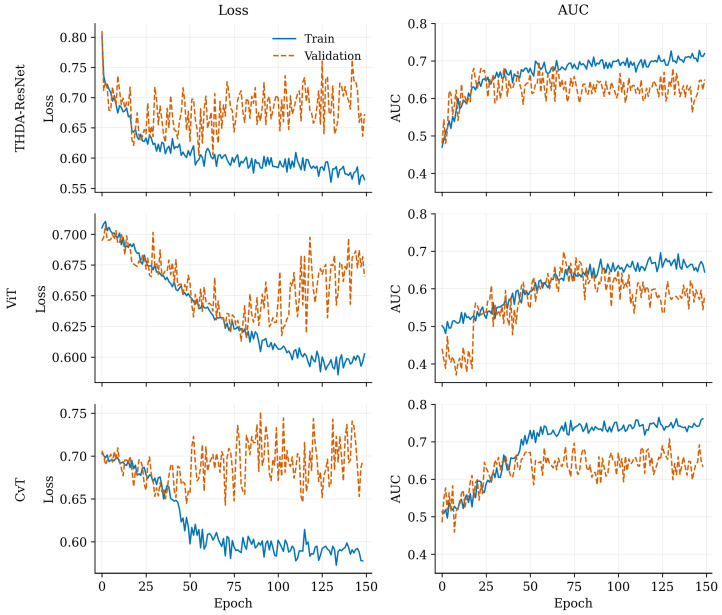
Training and validation curves over epochs for the three architectures (THDA-ResNet, ViT, CvT). Left column: loss; right column: AUC. Solid lines denote training and dashed lines validation. Metrics are computed at the slice level.

Models were fine-tuned using validation-based early stopping, retaining the checkpoint that achieved the best validation performance. Training was organized into successive rounds: when validation metrics failed to improve within a patience window of 20 epochs, the current round was terminated and the next round initialized from the best saved checkpoint rather than from the last epoch. This continue-from-best scheme was applied across all rounds, and the checkpoint with the highest validation performance overall was used for all reported evaluations. Figure 4 shows the training and validation curves for the three architectures. In all models the training loss decreased steadily while the validation loss declined early and then plateaued, indicating convergence without pronounced overfitting. THDA-ResNet reached the highest validation AUC and maintained the widest train–validation margin, consistent with its stronger discrimination on the test set. ViT and CvT converged more slowly and to lower validation AUC, with CvT showing the largest gap between its rising training AUC and a flatter validation AUC, indicating weaker generalization at this data scale. These curves are computed at the slice level, whereas all reported test-set results are at the patient level after aggregation; because tumor-bearing slices differ in tumor burden, slice-level validation metrics can be lower than the corresponding patient-level test metrics.

### 3.4. Patient-Level Prediction and Operating Point

Slice-level prediction scores were aggregated to a patient-level score using the median, selected on the validation set.

We report results at two thresholds: the conventional default of 0.5, and a prevalence-adjusted operating point of 0.7. Reporting the 0.5 threshold ensures comparability with standard practice, while the 0.7 threshold addresses a specific property of our training setup. Because training mini-batches were class-balanced at a 50:50 ratio to stabilize optimization, while HER2-positive lesions represent only about 24% of the cohort, models calibrated on these balanced batches over-predict the positive class at the 0.5 cut-off, inflating false positives; raising the operating point to 0.7 counteracts this sampling bias and realigns the decision boundary with the true population prevalence. The value was not arbitrary: across validation-set sweeps, the optimal threshold for every model and preprocessing configuration fell consistently between 0.65 and 0.75, and we adopted a single shared value from this range to evaluate all configurations under one transparent, deployable decision rule rather than tuning a separate cut-off per configuration. As AUC, our primary comparison metric, is threshold-independent, the model ranking is unaffected by either choice.

### 3.5. External Validation

Trained models were evaluated on the external BreastDCEDL_AMBL cohort described above, using the same input construction, preprocessing, and patient-level aggregation as for the I-SPY test set. No training, fine-tuning, or hyperparameter adjustment was performed on AMBL data.

### 3.6. Statistical Analysis

Performance metrics included accuracy (ACC), area under the receiver operating characteristic curve (AUC), sensitivity, specificity, positive predictive value (PPV), and negative predictive value (NPV). AUC 95% confidence intervals (CI) were obtained by stratified bootstrap (1000 resamples), sampling positive and negative cases independently to preserve class prevalence in each replicate. Pairwise comparisons of AUC between models were performed using DeLong’s test [39] on the best-performing preprocessing strategy of each model on the I-SPY test set. Reported *p*-values are two-sided; p<0.05 was considered statistically significant.

This study constituted a secondary analysis of fully de-identified, publicly available datasets from The Cancer Imaging Archive; institutional review board approval was therefore not required at the authors’ institution.

## 4. Results

### 4.1. Model Performance Comparison

THDA-ResNet achieved the highest AUC for HER2 prediction at threshold 0.5 (Table 3, which reports the full performance metrics and confusion-matrix counts for each preprocessing strategy). THDA-ResNet AUCs ranged from 0.69 to 0.74 across preprocessing strategies (accuracy 0.39–0.58), while ViT achieved AUCs of 0.62–0.66 (accuracy 0.67–0.72) and CvT achieved AUCs of 0.60–0.63 (accuracy 0.66–0.70), as shown in Table 4 (full metrics and confusion-matrix counts per preprocessing strategy). Despite transformers showing higher accuracy values, their lower AUCs and sensitivity (0.28–0.56 for transformers vs. 0.78–0.94 for THDA-ResNet) indicate they reached their accuracy by favoring specificity at the cost of missing positive cases.

For THDA-ResNet, channel-wise upper-clipping strategies ($q_{0.99}$, $q_{0.98}$, $q_{0.95}$) consistently yielded the best performance. N4 bias correction did not improve performance and was therefore excluded from transformer evaluations and subsequent analyses.

To assess whether the observed AUC differences between models were statistically significant, we performed pairwise DeLong tests on the best-performing preprocessing strategy of each model on the full I-SPY test set. THDA-ResNet (channel upper clipping $q_{0.98}$, AUC 0.74) achieved a higher AUC than CvT (global min–max, AUC 0.63), with the difference reaching borderline significance (p=0.054); the difference relative to ViT (per-slice min–max, AUC 0.66) did not reach statistical significance (p=0.14), reflecting the limited size of the test cohort. ViT and CvT did not differ significantly from each other (p=0.60).

The contrast between models becomes more visible at threshold 0.7. The best THDA-ResNet configuration (channel upper clipping $q_{0.98}$) reached accuracy 0.75 with sensitivity 0.41 and specificity 0.86, retaining nontrivial discrimination. In contrast, the best ViT and CvT configurations collapsed to near-trivial classifiers (sensitivity 0.00 and 0.03, respectively, specificity 1.00 and 0.97), achieving their accuracy almost entirely by predicting the majority class. THDA-ResNet was therefore the only architecture that produced usable predictions at this operating point.

### 4.2. Optimal THDA-ResNet Configuration

Table 5 presents the top-performing THDA-ResNet configurations using upper-clipping normalization (no N4 correction) at the validation-derived operating threshold of 0.7, together with their confusion-matrix counts. Channel upper clipping at $q_{0.98}$, $q_{0.95}$, $q_{0.99}$, and global upper clipping at $q_{0.99}$ all achieved accuracies of 0.72–0.75 with AUCs of 0.73–0.74 on the complete test cohort. Performance on the I-SPY 2 subset was slightly higher, with accuracies of 0.74–0.78 and AUCs of 0.72–0.75.

Channel upper clipping at $q_{0.98}$ yielded the best overall performance: accuracy 0.75, AUC 0.74 (95% CI 0.65–0.83), sensitivity 0.41, and specificity 0.86 on the full test set. Compared with the conventional default threshold of 0.5, the threshold of 0.7 substantially improved specificity (0.81–0.87 vs. 0.30–0.51) at the cost of sensitivity (0.31–0.47 vs. 0.78–0.94), resulting in better-balanced predictions for clinical decision support.

### 4.3. External Validation on BreastDCEDL_AMBL Dataset

To assess model generalizability beyond the I-SPY trials, we evaluated the trained THDA-ResNet on the external BreastDCEDL_AMBL cohort described above. The same I-SPY-trained model was applied directly, with no fine-tuning, using the median patient-level aggregation and the threshold of 0.7 selected on the I-SPY validation set.

Table 6 presents external validation performance across the same preprocessing strategies evaluated on I-SPY. Global min–max normalization yielded the highest overall performance with accuracy 0.67 and AUC 0.66 (95% CI 0.49–0.81), and balanced sensitivity (0.56) and specificity (0.74). Per-slice min–max and lower quantile clipping performed comparably (AUC 0.66, accuracy 0.65). Among the upper-clipping strategies, channel upper clipping at $q_{0.98}$ produced a more clinically balanced operating point on AMBL (sensitivity 0.69, specificity 0.63, NPV 0.77) at the cost of a lower AUC (0.61). Other upper-clipping methods, which were the best-performing on I-SPY, were less competitive on AMBL.

Performance on this external dataset was lower than on the I-SPY test data (AUC 0.60–0.66 vs. 0.73–0.74), reflecting expected domain shift between institutions with different scanner vendors, field strengths, imaging protocols, and patient populations. The 95% confidence intervals were correspondingly wide due to the smaller external cohort (43 lesions). Despite this gap, the model maintained discriminative ability above chance, with most preprocessing strategies producing AUCs around 0.60–0.66 and the best configuration achieving balanced sensitivity and specificity at the I-SPY-derived operating point. The relative ranking of preprocessing strategies was not preserved across cohorts: min–max-based methods performed best on AMBL, whereas channel upper clipping was best on I-SPY, suggesting that intensity normalization should be revisited when generalizing to new institutions.

## 5. Discussion

We systematically evaluated preprocessing strategies and deep learning architectures for HER2 prediction from DCE-MRI using a multicenter cohort (*n* = 1149) and an independent external test cohort. THDA-ResNet achieved the highest AUC across all preprocessing strategies, and preprocessing choice had a substantial effect on performance for every architecture.

Why HER2 and the choice of receptor targets.

Clinical breast cancer treatment is guided by the combined receptor profile (estrogen receptor, progesterone receptor, and HER2), and a model that predicts a single receptor in isolation cannot replace standard pathological assessment. Our motivation here was different. We sought to test whether individual receptor status has spatial and temporal signatures in DCE-MRI that a deep network can extract, treating each receptor as a separate imaging-biomarker question rather than as a step toward replacing biopsy. HER2 was the primary target of this manuscript because HER2-targeted therapies are among the most specific and impactful subtype-driven treatment decisions in breast cancer [3,4], and because non-invasive HER2 prediction has direct value as an adjunct decision-support signal at staging and during therapy. We frame this work as evaluating the achievable performance of imaging-only HER2 prediction, not as a candidate replacement for pathology. The hormone-receptor question is left open as future work: I-SPY trial metadata provide only the combined hormone-receptor (HR) status (the union of ER and PR) rather than separate ER and PR labels, which limits direct comparison with studies predicting ER and PR individually; testing whether the same THDA-ResNet pipeline can extract a hormone-receptor signal from DCE-MRI is a natural next step using the same dataset.

N4 bias-field correction is unnecessary.

Across nearly all experiments, N4 bias-field correction [20]—a standard step in MRI radiomics pipelines—did not improve performance and in several configurations slightly degraded it. This finding has practical implications beyond accuracy: N4 correction is computationally expensive and typically requires resampling to an isotropic voxel grid. Removing it from the pipeline therefore yields a substantial reduction in preprocessing time, eliminates a parameter-laden step that varies across implementations, and improves reproducibility and protocol consistency between institutions. More broadly, our results suggest that resampling-based preprocessing conventions inherited from radiomics workflows do not transfer directly to deep learning pipelines and may be safely omitted.

Intensity normalization trade-offs.

Different normalization strategies present distinct trade-offs. Per-slice normalization maximizes local contrast within each slice but disrupts spatial intensity relationships across the volume. Per-channel normalization maximizes contrast within each temporal phase but corrupts temporal relationships between phases. Global normalization preserves both spatial and temporal relationships but severely compresses dynamic range due to long-tailed intensity distributions. On I-SPY, channel-wise upper-quantile clipping was the most effective strategy. On AMBL, simple min–max normalization performed best, while channel upper clipping at $q_{0.98}$ produced the most clinically balanced operating point. The fact that the optimal preprocessing strategy did not transfer cleanly across cohorts indicates that intensity normalization remains an open question for cross-institutional generalization, and that a single preprocessing recipe should not be assumed to be universally optimal.

CNN versus transformer comparison.

THDA-ResNet achieved a higher AUC than both transformer baselines (0.74 vs. ViT 0.66 and CvT 0.63) under matched experimental conditions: all three models were initialized with ImageNet-pretrained weights, fine-tuned on the same training data with identical preprocessing, and evaluated with the same patient-level aggregation. The difference reached borderline significance versus CvT (p=0.054) but not versus ViT (p=0.14), reflecting the limited size of the test cohort. We therefore frame THDA-ResNet as a more reliable default at this data scale rather than as significantly superior to ViT on AUC. The contrast became more visible at threshold 0.7: THDA-ResNet retained nontrivial discrimination (sensitivity 0.41, specificity 0.86), while ViT and CvT collapsed to near-trivial classifiers (sensitivity 0.00 and 0.03, specificity 1.00 and 0.97), achieving their accuracy almost entirely by predicting the majority class. We interpret these results not as evidence that CNNs are intrinsically superior, but as a reflection of dataset size: transformer-based architectures typically require substantially larger training datasets to outperform CNNs [9], and we expect that on much larger HER2 cohorts transformers will eventually take the lead. For the medium-sized public datasets currently available for HER2 prediction, however, a CNN backbone with appropriate attention mechanisms produces a more reliable predictor. The architectural design of THDA-ResNet—processing each temporal phase through a separate head before fusion, rather than channel-concatenating phases at the input—may also contribute to its sample efficiency by preserving phase-specific features explicitly. Hybrid CNN–transformer designs [10] are a natural direction for future work.

Cross-institutional generalization.

External validation on the AMBL cohort (AUC 0.60–0.66 across preprocessing methods) was lower than on I-SPY (AUC 0.73–0.74), reflecting expected domain shift between institutions with different scanner vendors, field strengths, imaging protocols, and patient populations. Despite this gap and the wide confidence intervals expected from a 43-lesion cohort, the model retained discriminative ability above chance and produced clinically reasonable sensitivity-specificity trade-offs at the I-SPY-derived operating point. Compared with prior multicenter deep learning studies, our results are competitive: Chen et al. [29] reported AUC 0.63 on I-SPY2 and 0.56 on cross-cohort validation, and Shiri et al. [31] reported AUC 0.70 on Duke (*n* = 922).

Error analysis.

To characterize model errors clinically, we evaluated each model at its own threshold (maximizing Youden’s index) and cross-referenced the misclassified cases with the available clinicopathologic metadata. Across all three architectures, false positives were dominated by HR-positive/HER2-negative (luminal) and triple-negative tumors (THDA-ResNet 28 and 20; ViT 15 and 8; CvT 13 and 8), while false negatives skewed toward HR-positive/HER2-positive co-positive lesions (THDA-ResNet 4 of 4; ViT 9 of 14; CvT 9 of 16). The misclassifications therefore concentrated in biologically ambiguous overlap zones—HER2-positive tumors co-expressing hormone receptors, and HER2-negative tumors with aggressive enhancement such as triple-negative disease—consistently across CNN and transformer models, rather than reflecting architecture-specific failure modes. Tumor volume did not separate misclassified from correctly classified lesions. Of the 32 HER2-positive lesions, 10 were correctly identified by all three models and 2 were missed by all three, indicating a shared set of easy and hard cases with model-specific differences between them. Because ground-truth HER2 status in these cohorts derives from pathology (immunohistochemistry/in situ hybridization) rather than radiologist interpretation, a reader-level assessment of diagnostic ambiguity was not possible in the present data and remains an objective for future reader studies.

Methodological transparency.

The pipeline relies only on publicly released data: the I-SPY temporal-phase indices and tumor segmentations are part of the BreastDCEDL release [11], and the AMBL segmentations are part of the source TCIA collection [22]. Importantly, segmentations are used in our pipeline only to identify which axial slices contain tumor; they are not consumed as a model input, and segmentation accuracy is therefore not a strict requirement. A fully automated pipeline can be built by replacing the released masks with an off-the-shelf breast lesion segmenter such as the U-Net ensemble of Khaled et al. [37].

Limitations.

Several limitations warrant discussion. Our 2D approach does not exploit volumetric structure. We focused exclusively on DCE sequences, whereas multiparametric models incorporating T2-weighted and diffusion-weighted imaging could improve performance. We evaluated binary HER2 classification, though ordinal approaches could better capture HER2-low cases. Sensitivity at the I-SPY-derived operating point remains modest (0.41 on the internal cohort), reflecting the class imbalance of the training data (24% HER2-positive) and indicating that the present model is best framed as an adjunct decision-support tool rather than a standalone diagnostic. The external validation cohort is small (43 lesions) and from a single institution, producing wide confidence intervals; broader external validation across multiple institutions remains essential. Finally, while attention-map analysis could inform radiological interpretation, validation is needed to determine whether learned representations correspond to known biological characteristics of HER2-positive disease.

## 6. Conclusions

A Triple-Head Dual-Attention ResNet captures spatiotemporal features in DCE-MRI for HER2 prediction and generalizes reasonably to an independent external cohort. Two practical conclusions follow. First, N4 bias-field correction can be omitted from the preprocessing pipeline without harming performance, and in some configurations omitting it slightly improves performance; this yields a substantial reduction in preprocessing time and improves reproducibility and protocol consistency between institutions. Second, on medium-sized public DCE-MRI cohorts of the scale currently available for HER2 prediction, an ImageNet-pretrained CNN backbone with multi-phase attention fusion produces a more reliable predictor than ImageNet-pretrained transformer baselines under matched conditions. We expect this gap to narrow and likely reverse as substantially larger HER2 cohorts become available, but for the data scales presently accessible to most research groups, attention-driven CNNs remain a strong default choice. These generalizability claims are tempered by the small external cohort (43 lesions, single institution), which yields wide confidence intervals; evaluation on larger, multi-institutional external datasets remains necessary before clinical use.

## Figures and Tables

**Table 1:** I-SPYtrial patient demographics and clinical characteristics. Data are presented as *n* (%) unless otherwise specified.

Characteristic	Training	Testing	Validation	Total
Number of patients	885	132	132	1149
Age, mean ± SD (years)	50.1 ± 10.5	50.5 ± 10.5	50.4 ± 10.8	50.2 ± 10.5
**Race, *n* (%)**				
White	662 (74.8)	96 (72.7)	96 (72.7)	854 (74.3)
Black	139 (15.7)	25 (18.9)	25 (18.9)	189 (16.4)
Other/Unknown	84 (9.5)	11 (8.3)	11 (8.3)	106 (9.2)
**Biomarkers/Response, *n* (%)**				
HR-positive	556 (62.8)	86 (65.2)	85 (64.4)	727 (63.3)
HER2-positive	213 (24.1)	32 (24.2)	34 (25.8)	279 (24.3)
pCR achieved	234 (26.4)	29 (22.0)	29 (22.0)	292 (25.4)

HR, hormone receptor; HER2, human epidermal growth factor receptor 2; pCR, pathologic complete response; SD, standard deviation.

**Table 2:** Training and implementation hyperparameters for THDA-ResNet and the transformer baselines.

Setting	THDA-ResNet	ViT	CvT
**Architecture and initialization**			
Backbone/base model	ResNet34	ViT-MAE-Base	CvT-13
Source weights	torchvision ResNet34	facebook/vit-mae-base	microsoft/cvt-13
ImageNet pretraining	Supervised	Self-supervised (MAE)	Supervised
Input size	224×224	224×224	224×224
**Optimization**			
Optimizer	AdamW	AdamW	AdamW
Loss	Cross-entropy	Cross-entropy	Cross-entropy
Batch size	16	16	16
Max epochs	150	150	150
Early-stopping patience	20	20	20
**Learning-rate schedule (constant plateau, then cosine decay to floor)**			
Base learning rate	$1\times 10^{-4}$	$5\times 10^{-6}$	$5\times 10^{-6}$
Floor learning rate	$2\times 10^{-6}$	$2\times 10^{-7}$	$2\times 10^{-7}$
Plateau length (epochs)	2	4	4
Decay length (epochs)	40	50	50
**Regularization (dropout)**			
Main dropout	classifier 0.5, fusion 0.25	hidden 0.2	drop/drop-path 0.2
Attention dropout	0.1	0.2	0.1

The learning-rate schedule applies a short constant plateau (plateau length) followed by cosine decay (decay length) from the base to the floor learning rate. Dropout values denote the rate at each location: for THDA-ResNet, the classifier head (0.5), the temporal feature-fusion layer (0.25), and the spatial attention modules (0.1); for ViT, the hidden and attention layers (0.2 each); and for CvT, the stochastic-depth/drop-path and attention layers (0.2 and 0.1). Early-stopping patience is the number of epochs without validation improvement after which training is stopped and the best checkpoint restored.

**Table 3:** HER2 prediction performance of the Triple-Head Dual-Attention ResNet across preprocessing methods at threshold 0.5.

Preprocessing	ACC	AUC (95% CI)	Sens	Spec	PPV	NPV	TP	TN	FP	FN
Per-slice min–max	0.568	0.723 (0.62–0.81)	0.781	0.500	0.333	0.877	25	50	50	7
Global min–max	0.462	0.700 (0.60–0.80)	0.906	0.320	0.299	0.914	29	32	68	3
Lower quantile clipping ($q_{0.10}$)	0.568	0.720 (0.62–0.81)	0.781	0.500	0.333	0.877	25	50	50	7
Global upper clipping ($q_{0.99}$)	0.500	0.726 (0.63–0.82)	0.938	0.360	0.319	0.947	30	36	64	2
Channel upper clipping ($q_{0.99}$)	0.515	0.731 (0.63–0.82)	0.844	0.410	0.314	0.891	27	41	59	5
**Channel upper clipping ($q_{0.98}$)**	**0.523**	**0.744 (0.65–0.83)**	**0.875**	**0.410**	**0.322**	**0.911**	**28**	**41**	**59**	**4**
Channel upper clipping ($q_{0.95}$)	0.455	0.734 (0.64–0.81)	0.938	0.300	0.300	0.938	30	30	70	2
N4 Per-slice min–max	0.568	0.719 (0.62–0.81)	0.781	0.500	0.333	0.877	25	50	50	7
N4 Global min–max	0.485	0.710 (0.60–0.80)	0.938	0.340	0.312	0.944	30	34	66	2
N4 Lower quantile clipping ($q_{0.10}$)	0.576	0.722 (0.63–0.82)	0.781	0.510	0.338	0.879	25	51	49	7
N4 Global upper clipping ($q_{0.99}$)	0.500	0.687 (0.58–0.78)	0.906	0.370	0.315	0.925	29	37	63	3
N4 Channel upper clipping ($q_{0.99}$)	0.508	0.698 (0.60–0.79)	0.906	0.380	0.319	0.927	29	38	62	3
N4 Channel upper clipping ($q_{0.98}$)	0.523	0.712 (0.62–0.79)	0.875	0.410	0.322	0.911	28	41	59	4
N4 Channel upper clipping ($q_{0.95}$)	0.394	0.686 (0.59–0.78)	0.938	0.220	0.278	0.917	30	22	78	2

ACC, accuracy; AUC, area under the receiver operating characteristic curve; Sens, sensitivity; Spec, specificity; PPV, positive predictive value; NPV, negative predictive value; TP, true positives; TN, true negatives; FP, false positives; FN, false negatives. The four columns TP, TN, FP, and FN together constitute the confusion matrix for each configuration: TP and TN are the correctly classified HER2-positive and HER2-negative cases, while FP and FN are the misclassified cases (HER2-negative predicted as positive, and HER2-positive predicted as negative, respectively). Slice-level scores were aggregated to patient-level scores using the median. AUC 95% confidence intervals were computed by stratified bootstrap (1000 resamples). The best-performing configuration (highest AUC) is highlighted in bold.

**Table 4:** HER2 prediction performance of the ViT and CvT transformer baselines across preprocessing methods at threshold 0.5, without N4 correction.

Preprocessing	ACC	AUC (95% CI)	Sens	Spec	PPV	NPV	TP	TN	FP	FN
**ViT Transformer**										
**Per-slice min–max**	**0.720**	**0.659 (0.56–0.76)**	**0.562**	**0.770**	**0.439**	**0.846**	**18**	**77**	**23**	**14**
Global min–max	0.674	0.643 (0.53–0.75)	0.500	0.730	0.372	0.820	16	73	27	16
Lower quantile clipping ($q_{0.10}$)	0.720	0.659 (0.56–0.76)	0.562	0.770	0.439	0.846	18	77	23	14
Global upper clipping ($q_{0.99}$)	0.682	0.626 (0.51–0.74)	0.406	0.770	0.361	0.802	13	77	23	19
Channel upper clipping ($q_{0.99}$)	0.667	0.629 (0.52–0.74)	0.500	0.720	0.364	0.818	16	72	28	16
Channel upper clipping ($q_{0.98}$)	0.667	0.624 (0.51–0.73)	0.469	0.730	0.357	0.811	15	73	27	17
**CvT Transformer**										
Per-slice min–max	0.659	0.607 (0.49–0.72)	0.375	0.750	0.324	0.789	12	75	25	20
**Global min–max**	**0.659**	**0.626 (0.49–0.74)**	**0.531**	**0.700**	**0.362**	**0.824**	**17**	**70**	**30**	**15**
Lower quantile clipping ($q_{0.10}$)	0.667	0.610 (0.49–0.72)	0.406	0.750	0.342	0.798	13	75	25	19
Channel upper clipping ($q_{0.99}$)	0.689	0.596 (0.48–0.72)	0.344	0.800	0.355	0.792	11	80	20	21
Channel upper clipping ($q_{0.98}$)	0.674	0.598 (0.49–0.71)	0.406	0.760	0.351	0.800	13	76	24	19
Channel upper clipping ($q_{0.95}$)	0.697	0.615 (0.49–0.72)	0.281	0.830	0.346	0.783	9	83	17	23

ACC, accuracy; AUC, area under the receiver operating characteristic curve; Sens, sensitivity; Spec, specificity; PPV, positive predictive value; NPV, negative predictive value; TP, true positives; TN, true negatives; FP, false positives; FN, false negatives. The four columns TP, TN, FP, and FN together constitute the confusion matrix for each configuration: TP and TN are the correctly classified HER2-positive and HER2-negative cases, while FP and FN are the misclassified cases (HER2-negative predicted as positive, and HER2-positive predicted as negative, respectively). Slice-level scores were aggregated to patient-level scores using the median. AUC 95% confidence intervals were computed by stratified bootstrap (1000 resamples). The best-performing configuration (highest AUC) within each model is highlighted in bold.

**Table 5:** Top THDA-ResNet configurations at threshold 0.7 (upper clipping, no N4 correction).

**Complete test cohort—132 cases**										
**Preprocessing**	**ACC**	**AUC (95% CI)**	**Sens**	**Spec**	**PPV**	**NPV**	**TP**	**TN**	**FP**	**FN**
**Channel upper clipping ($q_{0.98}$)**	**0.750**	**0.744 (0.65–0.83)**	**0.406**	**0.860**	**0.481**	**0.819**	**13**	**86**	**14**	**19**
Channel upper clipping ($q_{0.95}$)	0.720	0.734 (0.64–0.82)	0.406	0.820	0.419	0.812	13	82	18	19
Channel upper clipping ($q_{0.99}$)	0.735	0.731 (0.64–0.82)	0.312	0.870	0.435	0.798	10	87	13	22
Global upper clipping ($q_{0.99}$)	0.727	0.726 (0.64–0.81)	0.469	0.810	0.441	0.827	15	81	19	17
**I-SPY 2 test cohort—99 cases**										
**Preprocessing**	**ACC**	**AUC (95% CI)**	**Sens**	**Spec**	**PPV**	**NPV**	**TP**	**TN**	**FP**	**FN**
**Channel upper clipping ($q_{0.98}$)**	**0.778**	**0.747 (0.64–0.84)**	**0.364**	**0.896**	**0.500**	**0.831**	**8**	**69**	**8**	**14**
Channel upper clipping ($q_{0.95}$)	0.737	0.721 (0.61–0.83)	0.364	0.844	0.400	0.823	8	65	12	14
Channel upper clipping ($q_{0.99}$)	0.758	0.739 (0.63–0.83)	0.227	0.909	0.417	0.805	5	70	7	17
Global upper clipping ($q_{0.99}$)	0.768	0.746 (0.64–0.84)	0.409	0.870	0.474	0.838	9	67	10	13

Metric definitions are consistent with those in Table 3; the TP, TN, FP, and FN columns together constitute the confusion matrix for each configuration. AUC 95% confidence intervals were computed by stratified bootstrap (1000 resamples). No training or fine-tuning was performed on this dataset. The best-performing configuration (highest AUC) is highlighted in bold.

**Table 6:** External validation on the BreastDCEDL_AMBL dataset (42 patients, 43 lesions) using THDA-ResNet trained on I-SPY data, evaluated at threshold 0.7.

Preprocessing	ACC	AUC (95% CI)	Sens	Spec	PPV	NPV	TP	TN	FP	FN	N
Per-slice min–max	0.651	0.655 (0.47–0.83)	0.500	0.741	0.533	0.714	8	20	7	8	43
**Global min–max**	**0.674**	**0.664 (0.49–0.81)**	**0.562**	**0.741**	**0.562**	**0.741**	**9**	**20**	**7**	**7**	**43**
Lower quantile clipping ($q_{0.10}$)	0.651	0.655 (0.48–0.82)	0.500	0.741	0.533	0.714	8	20	7	8	43
Global upper clipping ($q_{0.99}$)	0.581	0.620 (0.43–0.79)	0.688	0.519	0.458	0.737	11	14	13	5	43
Channel upper clipping ($q_{0.99}$)	0.558	0.606 (0.42–0.78)	0.625	0.519	0.435	0.700	10	14	13	6	43
Channel upper clipping ($q_{0.98}$)	0.651	0.613 (0.43–0.79)	0.688	0.630	0.524	0.773	11	17	10	5	43
Channel upper clipping ($q_{0.95}$)	0.535	0.597 (0.41–0.78)	0.625	0.481	0.417	0.684	10	13	14	6	43

Metric definitions are consistent with those in Table 3. AUC 95% confidence intervals were computed by stratified bootstrap (1000 resamples). No training or fine-tuning was performed on this dataset. The best-performing configuration (highest AUC) is highlighted in bold.

## Data Availability

The datasets used in this study are publicly available. BreastDCEDL (I-SPY trials) [11]: https://doi.org/10.5281/zenodo.15627233. BreastDCEDL_AMBL [24] (88 patients, 132 lesions; HER2 status available for 42 patients, 43 lesions): https://doi.org/10.5281/zenodo.17189101. Original source data were obtained from TCIA: The AMBL collection, I-SPY 1, and I-SPY 2.

## Abbreviations

The following abbreviations are used in this manuscript:

ACC	Accuracy
AUC	Area under the receiver operating characteristic curve
CI	Confidence interval
CNN	Convolutional neural network
CvT	Convolutional Vision Transformer
DCE-MRI	Dynamic contrast-enhanced magnetic resonance imaging
HER2	Human epidermal growth factor receptor 2
HR	Hormone receptor
NPV	Negative predictive value
PPV	Positive predictive value
TCIA	The Cancer Imaging Archive
THDA-ResNet	Triple-Head Dual-Attention ResNet
ViT	Vision Transformer
